# A mediation model explaining the impact of fear of COVID-19 and COVID-19- induced changes in multiple life domains on adolescents’ subjective well-being in sub-Saharan Africa

**DOI:** 10.1371/journal.pone.0337188

**Published:** 2025-11-19

**Authors:** Evelyn Aboagye Addae, Moses Adjei, Uchechi Shirley Anaduaka, David Kyetuo Wuollah-Dire, Regobert Bondong

**Affiliations:** 1 Glasgow Caledonian University, GCU London, London, United Kingdom; 2 School of Science and the Environment, Memorial University, Newfoundland and Labrador, Canada; 3 School of Public Health, University of Alberta, Edmonton, Canada; 4 Nadowli Kaleo District Assembly, Nadowli, Ghana; 5 McCoy College of Education, Nadowli, Ghana; University of Jyvaskyla, FINLAND

## Abstract

Though the impact of COVID-19 pandemic’s effects on individuals’ life domains and quality of life has been widely researched, there remains unanswered questions on the mechanisms that explain the impact of fear of COVID-19 on different measurements of adolescents’ subjective well-being (SWB) particularly in the sub-Saharan African context. In a mediation model, we employed data collected during the pandemic to examine the mediating mechanisms that links fear of COVID-19 and COVID-19-induced changes in multiple life domains (subjective feelings of unsafety, positive affect and peer relationships) to different measurements of adolescents’ SWB (overall life satisfaction, overall happiness, subjective happiness, and composite SWB). Findings revealed significant negative correlations between fear of COVID-19 and all employed measurements of SWB as well as between the proposed mediating variables – changes in peer relationship, positive affect, subjective feelings of unsafety and the different measurements of SWB. Adolescents who reported negative changes in peer relationship, positive affect and subjective feelings of unsafety were more likely to report poorer SWB including lower life satisfaction and happiness. For instance, adolescents who experienced increase in peer relationship were more likely to experience higher overall life satisfaction (*B* = .169, *p* < .005) compared to those who experienced a decrease in peer relationship. Also, adolescents who reported an increase in positive affect were more likely to report higher overall life satisfaction (*B* = .172, *p* < .005), overall happiness (*B* = .056, *p* < .005), composite SWB (*B* = .416, *p* < .005) and subjective happiness (*B* = .381, *p* < .001) while adolescents who reported a decrease in perceived safety were more likely to report lower composite SWB (*B* = −1.350, *p* < .001) compared to those who experienced an increase in perceived safety. While there was a significant direct negative effect of fear of COVID-19 on SWB in the absence of the mediators, there was no significant negative effect of fear of COVID-19 on SWB after adjusting for the mediators in the model. Consequently, the direct effect of fear of COVID-19 on all the measurements of SWB including overall life satisfaction and happiness were completely mediated by changes in peer relationship, positive affect and subjective feelings of unsafety; suggesting indirect effects of fear of COVID-19 on the adolescents’ SWB. For example, change in peer relationship and positive affect mediated the relationship between fear of COVID-19 and overall life satisfaction. Again, change in positive affect and change in subjective feeling of unsafety significantly mediated the effect of fear of COVID-19 on composite SWB and only change in positive affect mediated the effect of fear of COVID-19 on subjective happiness. The mechanism explaining the relationship between fear of COVID-19 and COVID-19-induced changes in multiple life domains and adolescents SWB and the implications for public health policy are discussed.

## 1. Introduction

The COVID-19 pandemic globally induced several negative psychological responses in people including fear of COVID-19, disrupted multiple life domains and consequently impacted individuals’ quality of life including subjective well-being [1–6]; heightening existing psychosocial inequality in health and well-being. The consequences of these impacts range from short, medium, and long term, leaving some of the affected with risks of cognitive and mental health problems and possible trauma from the loss of intimate relationships and emotional well-being [6,7]. For young people particularly, the contagious nature of the virus and the associated death in the early phase of the pandemic sent waves of panic among this population group, exposing them to fear and anxiety irrespective of been infected by the virus or not (2, 3, 8–11). COVID-19 mitigation measures employed at national and global levels also induced fear and significant changes in multiple life domains of young people including their social relationships, feelings of safety, affect (emotions) and SWB [6,7,12–14]. Fear of COVID-19 and induced changes in multiple life domains have been linked to poorer quality of live and poorer SWB among diverse population groups including adolescents [6,7,13].

Adolescents are, particularly, crucial population group whose quality of life during the pandemic should exclusively be investigated due to the high vulnerability of this developmental stage to cognitive, social and emotional impacts of internal and external life events or circumstances [7,15]. For example, adolescents are vulnerable to mental health and social problems including risk taking behaviours which can have a negative impact on their adult lives [15]. Adolescence comprises of biological, psychological and social changes, and adversative psychological experiences (e.g., fear of COVID-19) and changing social environments (e.g., negative COVID-19 induced changes in peer relationship, feelings of safety and affect) are some of the risk factors that can render the developing adolescent brain and mind vulnerable to poor SWB [15]. This is because, the brain experiences significant transformation including cognitive changes in adolescence, involving synaptic pruning and myelination which makes adolescents more susceptible to both positive and negative environmental subjections [15]. Studies from around the world infer that, young people were vulnerable to fear of COVID-19 due to for example, fear of catching or spreading the virus and fear of COVID-induced lockdowns and quarantine [6,7,16]. However, contrasting findings on the impact of fear of COVID-19 exist, with some suggesting greater fear of COVID-19 in younger adults and others reporting no age differences [10]. Moreover, while several studies have reported indirect impact of fear of COVID-19 on different measurements of adults’ quality of life [e.g., 4, 17], studies explaining the indirect impact of fear of COVID-19 on the SWB of adolescents remain generally scarce. There remain unanswered questions on the mechanisms that explain the impact of fear of COVID-19 on different measurements of adolescents’ SWB, particularly, amidst COVID-19 induced changes in domains of peer relationship, feelings of safety, and affect. Amassing knowledge and understanding the impacts of fear of COVID-19 and the mechanism by which it affected various domains of adolescents’ SWB during the pandemic is essential for promoting holistic national and global developmental programmes targeting positive adolescents’ development post-pandemic.

The impacts of COVID-19 on health and well-being have not been felt uniformly across society and population groups [6,7]. COVID-19 heightened existing structural and social inequalities, with especially negative health outcomes for those already disadvantaged in society [6,7]. For example, COVID-19 posed significant and unequal effects depending on age, where people live, their level of education, socioeconomic and health status [6,7]. This suggests a need for regional and age-specific studies on the impact of the pandemic’s effects on individuals. Such is even more important to help uncover the impact of the pandemic’s effects on the adolescent population in sub-Saharan Africa (SSA) where the world’s majority and fastest growing adolescent population are located [15] and adolescents’ health and well-being have been historically noted to be inequitable [5]. During the early years of the pandemic, the World Bank reported that the poor will suffer most from the COVID‐19 crisis, and that the pandemic could push about 49 million people including children and young people into extreme poverty in 2020. Significantly, low‐ and middle‐income countries (LMICs) were reported to be hugely affected with about half of the proposed new poor (23 million) been in SSA [3]. Again, early evidence from some SSA countries including Burkina Faso, Ethiopia, and Nigeria revealed that the pandemic has exerted enormous indirect impacts on multiple domains of adolescents’ lives, comprising inaccessibility to educational opportunities due to school closures which resulted in the loss of many crucial social protection roles of schools [5]. All this evidence revealed the plausible damaging mid‐ and long‐term impacts of COVID‐19 on adolescents in this region including broadened inequality gap in adolescent health, educational and psychological well‐being, and loss of social and human capital (protective health assets) which could pose detrimental life‐course consequences for adulthood [7]. Recognising that the world’s largest population, ‘adolescents’ live in LMICs infer that majority of the world’s young populace were at risk of the potential devastation of COVID‐19 including fear of COVID-19 (6). Providing empirical evidence on the impact of the pandemic on the life domains and SWB of adolescents from this region will thus reveal the consequences of the pandemic on a population group from a region that was projected to be at higher risk of the pandemic’s effects and offer novel insights into the various life domains of adolescents from SSA that were disrupted by the pandemic. This is vital for not only national regional stakeholders but also for global stakeholders interested in promoting healthy adolescent and young people post the pandemic in SSA.

Nevertheless, while empirical findings from SSA have revealed that COVID-induced disruptions in daily activities was associated with a higher prevalence of possible psychological distress, high anxiety level, and high depression level among adolescents in this region [5]; no study has yet examined the mechanism that links fear of COVID-19 and COVID-19 induced changes in multiple life domains to the SWB of adolescents in this region. It is hence vital that research on adolescents from SSA be encouraged to provide regional perspectives on the impact of the pandemic’s effects on adolescents’ outcomes. Most existing empirical studies investigating the indirect impact of fear of COVID-19 on individuals’ SWB have often been reported from countries beyond SSA countries including Ghana. Alas, none of these existing studies employed a mediation model to simultaneously explain the impact of fear of COVID-19 and COVID-19 induced changes in multiple life domains on varying measurements of adolescents’ SWB. Additionally, existing studies that focus specifically on adolescents are scarce because studies have often merged these age cohorts together with other age groups in studies on young people and adult populations [e.g., 4, 9, 10]. Nonetheless, among the groups identified as being most vulnerable during the pandemic are, among others, children and young people. Fears of infecting oneself has been identified as specific to the current pandemic in this age group [7,9], and a positive relationship has also been established between fear of COVID-19 and emotional problems in childhood and adolescence from the time of confinement onwards [7,11,13]. Also, adolescents and young adults appear to show higher levels of anxiety, depression and stress [9]. The present study therefore seeks to address these research gaps related to research on adolescents in SSA and answer the following research questions:

1. What were the impacts of COVID-19 pandemic’s effects (fear-of COVID-19 and COVID-19 induced changes in multiple life domains) on different domains of SWB and how did they vary by different measurement of SWB among adolescents in SSA?
2. What mechanism linked COVID-19 induced changes in multiple life domains to fear of COVID-19 and SWB among adolescents in SSA?
3. Could COVID-19 induced changes in multiple life domains mediate the impact of fear-of COVID-19 on different domains of SWB and how did they vary by different measurement of SWB among adolescents in SSA?

### 1.1. Impact of fear of COVID-19 on subjective well-being

Fear of COVID-19 involves concerns and anxiety relating to the COVID-19 pandemic [4,17]. While varying dimensions and measurements of SWB including life satisfaction and happiness of young people have been impacted by fear of COVID-19 across various regions [4,5,9,18], there remain gaps in studies that have simultaneously assessed the impacts of fear of COVID-19 on different measurements of SWB among the same adolescent populace. SWB is generally described as “an overarching concept regarding the quality of people’s lives” [19]. There exist two domains of SWB based on two theoretical components: (a) the ‘evaluative approach’ to SWB requiring individuals to cognitively assess their overall life or on detailed aspects of their life known as life satisfaction, and (b) the ‘hedonic approach’ requiring individuals to assess their feelings and emotions, happiness which is often utilised in global cognitive assessment of well-being [20,21]. A salient cognitive and affective component in measuring young people’s SWB is thus life satisfaction (LS) and happiness respectively [21]. During the pandemic, fear of COVID-19 was reported to significantly impact the life satisfaction and happiness of adults [4,17,22,23] with limited studies on adolescents. Most studies on young people have also either focused on life satisfaction or happiness without assessing both the evaluative and hedonic components of SWB pertaining to the same study participants. To account for the holistic assessment and multidimensionality of SWB, the present study takes both ‘evaluative’ and ‘hedonic’ approaches to examine the direct and indirect impact of fear of COVID-19 and COVID-19 induced changes in multiple life domains on different measurements of SWB including life satisfaction and happiness of adolescents in Ghana.

### 1.2. Impact of COVID-19-induced changes in multiple life domains on SWB

The pandemic induced significant changes in other multiple life domains of young people with domains of peer relationships, safety (security) and positive affect being hugely impacted [6,7,14]. These induced changes have been associated to poorer quality of life and SWB of young people including their life satisfaction and happiness during the pandemic [7,8,14]. According to the World Happiness Report, during the pande5mic affect (emotions) changed more than did life satisfaction [24]. Studies reported an increase in loneliness, depression, and distress during the pandemic [8,25] and while there was strengthening of closer ties, there was weakening of more distant relationships such as peer relationships across the world [26]. Again, while on average, life satisfaction, positive affect, and negative affect did not change significantly between December 2019 and March 2020, they decreased between March and May 2020 [25]. There was also a decrease in feelings of safety, increasing subjective feelings of unsafety (insecurity) among populations [6,7,12]. Indeed, negative changes in these life domains of young people are also risk factors for negative long-term health outcomes [7]. Feelings of unsafety has been proposed to be a huge source of distress due to its negative effects on life satisfaction and well-being. However, feelings of unsafety as a possible source of psychological distress that could impact the SWB of adolescents especially during the pandemic have mainly remained unnoticed in the literature [27]. Even though the literature on subjective safety (feelings of safety and unsafety) for the youngest cohorts has only developed recently compared to works examining adults’ experiences, today, evidence support the association between subjective feelings of unsafety and adolescents’ psychological well-being [27].

COVID-19 induced changes in life domains of peer relationship, safety and affect has bearing on future health consequences on individuals and the society at large. However, unlike the numerous studies reporting COVID-19-induced changes in different mental health measurements, there is less studies reporting COVID-19 induced changes in peer relationships, subjective feelings of unsafety and positive affect, especially, from SSA. It is hence critical to investigate changes that occurred in these domains during the pandemic and understand how fear of COVID-19 contributed to these induced changes in SSA. Understanding the potential for these induced changes in peer relationships, subjective feelings of unsafety and positive affect to function as risk and protective factors that could possibly mediate the relationship between fear of COVID-19 and the SWB of adolescents is desired for developing suitable programmes for resilience building and healthy adolescent population post-COVID-19 pandemic.

### 1.3. Proposed conceptual model and present study

The life event theory offers an understanding into the possible interplay that exist between fear of COVID-19, COVID-19 induced changes in multiple life domains and SWB. This theory is a notable framework in psychology that helps to understand the connection between major life events and their consequent impact on an individual’s psychological well-being [28]. It emphasizes the significance of understanding and addressing the role of life events in the development and management of psychological challenges. By realising the impacts of crucial life events, psychologists and therapists can offer suitable support and interventions to individuals experiencing emotional instability or other mental health problems [28]. The theory thus evaluates how diverse events, (e.g., marriage, divorce, job loss, or the death of a loved one) impacts a person’s mental health and overall quality of life. Such events are proposed to have an enormous impact on a person’s emotions, reasoning, and behaviours [28].

Life events can be categorised into positive and negative events. Positive events are often related to feelings of happiness, fulfillment, and an improved sense of well-being while negative events are often related to heightened stress, sadness, anxiety, and a decrease in psychological performance [28]. The life events theory which is similar to social stress theory suggest that an individual’s external environment influence well-being and thus proposes that significant life events can act as stressors, triggering emotional response and possibly leading to short-term or long-term changes in mental health. For example, life event studies revealed that higher stress related life events cause higher possibility of major depression and compulsive behaviour [28]. Similarly, many scholars have recognised the COVID-19 pandemic as a major negative life event that caused severe stress and posed severe negative impacts to the mental health and psychological well-being of individuals including children and adolescents [3,6,7]. Fear of COVID-19 is one of the direct psychological impacts of the pandemic and hence a major negative event that directly influenced various life domains and SWB of adolescents.

Moreover, the theory accentuates that the impact of life events on psychological well-being may vary depending on individual circumstances, prior experiences, and social support [28] which implies that the impact of fear of COVID-19 on the adolescents’ SWB may vary based on different life experiences during the pandemic. This supports the notion that coping strategies and other factors also play critical roles in how individuals respond and cope with life events [28] which can explain the possible varying impacts of fear of COVID-19 on adolescents’ SWB; thus, adolescents may have varying coping mechanisms and available support connections [28]. This theory is also supported by the Stress, Emotions, and Performance meta-model that posit that stressors arise from the environment an individual functions in, are mediated by the processes of cognitive appraisal and coping, and, consequently, individuals respond in diverse ways resulting in positive or negative stress responses [29]. In this process, some protective or risk factors mediate the effects of perceived stress on stress responses [29]. Similarly, we propose in this study that some adolescents may adapt well to fear of COVID-19 impacts through discovering protective factors that could help them to overcome negative effects of fear of COVID-19 (stressor) and sustain higher SWB (response), for example, through positive cognitive appraisal such as increase in peer relationship, positive affect and perceived safety (mediators). Others may struggle to cope, experiencing higher levels of distress and poorer SWB through decrease in peer relationship, positive affect and perceived safety during the pandemic. These induced changes in life domains during the pandemic could therefore function as coping strategies/mechanisms and risk factors or protective factors that could mediate the impact of fear of COVID-19 on adolescents’ SWB, suggesting indirect impact of fear of COVID-19 on SWB. Empirically, a systematic review investigating the indirect impact of the pandemic revealed significant indirect impacts of the pandemic on children and adolescents [16] suggesting the role of possible mediators in the relationship between the pandemic and SWB.

Similarly, scholars explaining theories of SWB suggest that SWB is mainly the outcome of several positive and negative events and circumstances in an individual’s life and that life events and changing circumstances can alter individuals’ short and long-term appraisal of their life satisfaction as a whole [21]. This supports the role of the pandemic as a major life event that could influence adolescents SWB as proposed by the life event theory. We hence proposed that the impact of fear of COVID-19 on adolescents’ SWB could be indirect and may vary depending on prior experiences of changes in life domains of safety, affect and peer relationships during the pandemic. We propose that fear of COVID-19 which was a major negative life event (stressor) could indirectly affect the SWB (stress response) of the adolescents through cognitive appraisal of their feelings of unsafety, positive affect and peer relationships (mediators). Subsequently, we propose that positive cognitive appraisal of changes in life domains of peer relationship, positive affect and subjective feelings of safety during the pandemic could function as protective factors/coping mechanisms (mediating mechanisms) that possibly protected the adolescents’ SWB against the negative impact of fear of COVID-19 (stressor).

Empirical studies supporting this theoretical argument imply that COVID-19 related emotional fear was found to significantly explain variability in life domains of “finances, loved ones, job, safety, school, mental health, physical health, social activities, and quality of life” [8]. Concerning possible indirect effect of fear of COVID-19 on SWB, several studies have found indirect effects of fear of COVID-19 on the life satisfaction of adults during the pandemic [4,17]. Nevertheless, studies that explore the indirect effect of fear of covid-19 on the SWB of adolescents especially amidst changes in life domains of safety, affect and peer relationships remain scarce.

**Fig 1:**
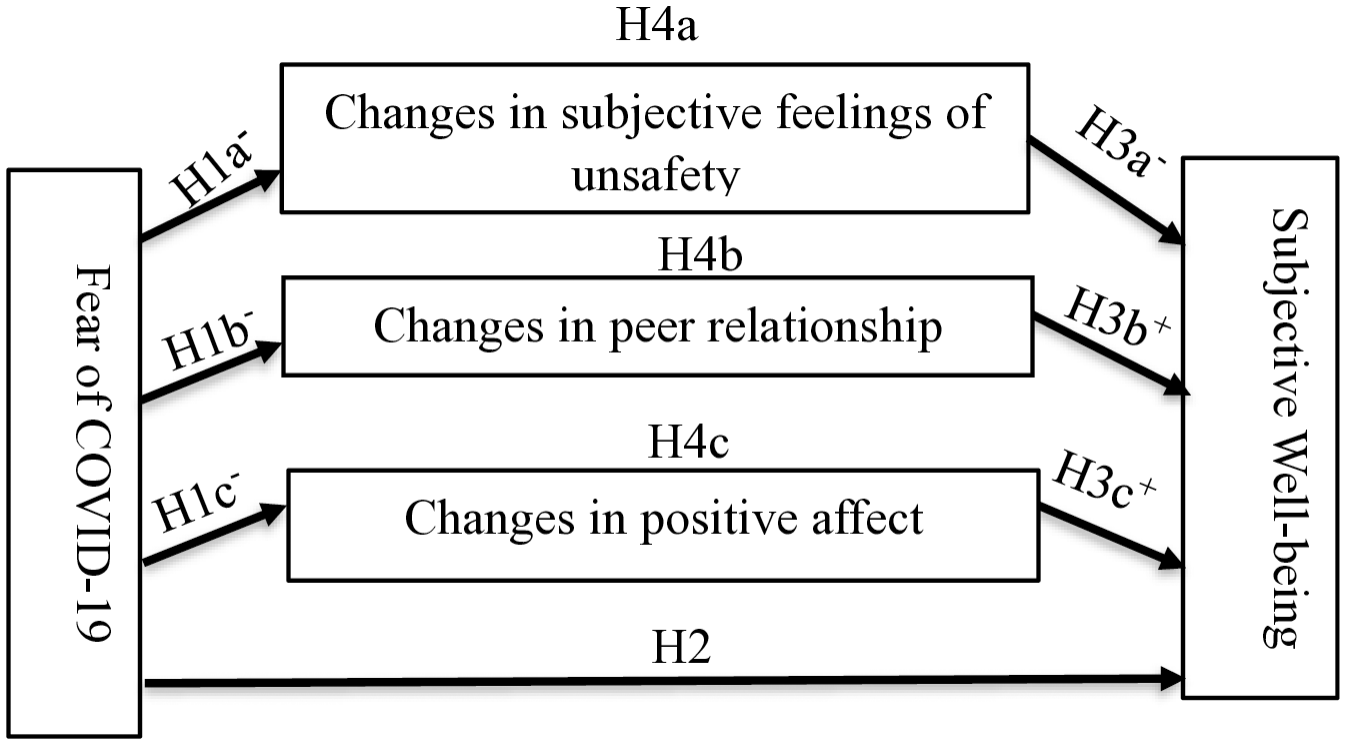
Hypothesised path analysis model for examining the interplay between fear of COVID-19, COVID-19 induced changes in multiple life domains and SWB. -  = negative effect, +  = positive effect.

In this study, we therefore examined COVID-19 induced changes in peer relationship, positive affect and subjective feelings of unsafety as mechanisms/mediators in the relationship between fear of COVID-19 and different measurements of SWB among adolescents in Ghana. A qualitative report by WHO [6] revealed that fear of the virus has been an unprecedented stressor to the mental health of many individuals in Ghana but quantitative studies that explains the relationship between fear of COVID-19 and the SWB od adolescents in Ghana remain scarce. This study aims to address the above research questions and provide empirical-quantitative evidence on (1) the impact of fear of COVID-19 and COVID-19 induced changes in life domains of safety focusing on ‘subjective feelings of unsafety’, intimacy focusing on ‘peer relationship’, and emotional well-being focusing on ‘positive affect’ on different measurements of SWB, (2) the mediating mechanism linking fear of COVID-19, COVID-19 induced changes in *subjective feelings of unsafety, peer relationship, positive affect* and SWB of adolescents and (3) the possible mediating role of *subjective feelings of unsafety, peer relationship, and positive affect* in the relationship between fear of COVID-19 and SWB of adolescents in Ghana. The measurements of the constructs utilised in the hypotheses have been presented at the method section. Drawing on the life event theory, Stress, Emotions, and Performance meta-model and findings from empirical research, we propose the following hypotheses as shown in Fig 1:

*Hypothesis 1: Fear of COVID-19 would negatively predict changes in subjective feelings of unsafety, peer relationship, and positive affect among adolescents during the pandemic.*

*Hypothesis 2: Fear of COVID-19 would negatively predict different measurements of adolescents’ SWB (H2a, H2b, H2c) during the pandemic.*

*Hypothesis 3: Changes in subjective feelings of unsafety, peer relationship, and positive affect would predict different measurements of adolescents’ SWB during the pandemic (H3a, H3b, H3c).*

*Hypothesis 4: Changes in subjective feelings of unsafety, peer relationship, and positive affect would mediate the relationship between fear of COVID-19 and SWB of adolescents during the pandemic (H4a, H4b, H4c).*

## 2. Materials and methods

### 2.1. Participants and procedure

This study was conducted during the COVID-19 pandemic, employed a cross-sectional school-based survey from 20^th^ June 2021–29^th^ July 2021 and collected data from 517 senior high school (SHS) adolescents (14–19yrs) selected from four SHS within four districts in the poorest region of Ghana, Upper West region to explore their experiences during the pandemic. It is a part of a broader project that investigated the impact of the pandemic on adolescents’ health, well-being, reproductive health, and access to healthcare in SSA. Utilising Ghana’s 2016 poverty mapping, the four districts were selected. Selecting these four districts was necessary to account for variations in economic conditions as well as cultural values of all adolescents in the region. In each district, the available secondary school was enlisted for the study. With the help of staff from the selected schools, qualified respondents were randomly selected from each of the four schools by stratifying eligible students within randomly selected classes based on the class sizes and randomised proportionally. The eligible students were provided with self-administered questionnaires on voluntary and anonymous bases for the data collection. The questionnaire was designed in English, the language of instruction in schools in Ghana. The researchers, who are natives of the region, offered interpretation of questions in the region’s main dialect on an individual basis. After the survey, focused group discussions were conducted to validate the participants’ understanding of the questionnaire.

The Committee on Human Research Publication and Ethics (CHRPE), School of Medical Sciences, Kwame Nkrumah University of Science and Technology and Komfo Anokye Teaching Hospital, Kumasi, Ghana (Ref: CHRPE/AP/211/21) provided the ethical approval for this study. Informed consent and approval were sought from the Ghana Education Service (GES) for the region -Wa (Ref: GES/UWR/WA77/ EP15/VOL/108) before engaging with all the selected schools. Approval letter from the GES was given to all Headmasters of the selected schools as a form of written informed consent from the regional authorities to seek their cooperation. All the selected participants (students) were in boarding schools living with strict COVID-19 mitigation protocols to keep them safe from spread of the virus during the survey. Therefore, verbal informed consent was also sought from the headmasters of the selected schools and the guardians of all the selected students before engaging the students in the survey. All verbal consents were documented in the researchers’ notes and witnessed by two of the researchers. Finally, written informed consent was sought from the participants to uphold their autonomy to participate in the survey. The participants signed written informed consent forms for documentation and were witnessed by their teachers before carrying out the survey. A full briefing was provided to the students on the research purpose, anonymity, confidentiality and publication of data, before the participants voluntarily signed written informed consent forms to participate in the study which took place in their schools. All other ethical protocols, including COVID-19 safety protocols were observed.

### 2.2. Measures

#### 2.2.1. Subjective Well-Being (SWB).

**Fig 2:**
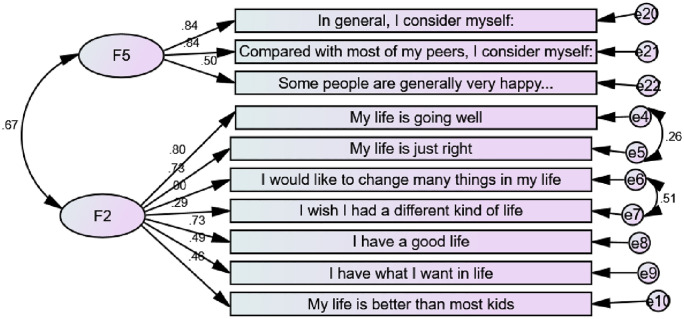
Constrained measurement model for subjective happiness (F5) and composite subjective well-being (F2) with standardised estimates.

Scholars have proposed varying scales for measuring SWB [30,31]. Four measurements of SWB were utilised; two cognitive dimensions: overall life satisfaction (OLS) (single-item scale) and composite subjective well-being (multiple-item scale) and two hedonic measures: overall happiness (single-item scale) and subjective happiness (multiple-item scale). OLS was assessed using the single-item Cantril ladder scale [30], which efficiently appraises the cognitive dimension of well-being. Participants selected one step out of 11 steps presented on an imaginary ladder on the questionnaire to indicate their OLS (10- highest satisfaction and 0– lowest satisfaction). Composite subjective well-being (CSWB), a cognitive measure was evaluated using the children world’s CSWB scale which consists of 7 questions about circumstances in their lives [33,34] (See Fig 2). The responses ranged from 1 ‘strongly disagree’ to 6 ‘strongly agree’. The participants were informed to think about how much they are satisfied with their life and how they are living now. The negative items were reverse coded for analysis, and the scale was reliable (α = .719).

Overall happiness was measured using a single-item scale adopted from the 2010–2012 World Value Survey [31]. The adolescents were asked, ‘Taking all things together, would you say you are…?’ Coded responses were 4 = ‘very happy,’ 3 = ‘rather happy,’ 2 = ‘not very happy,’ 1 = ‘not happy at all.’ Subjective happiness was assessed using a 4-item subjective happiness scale [32]. In this study, the fourth question reduced the reliability of the scale and was hence excluded during the scoring of the scales; thus a 3-item subjective happiness scale was employed in this study (see Fig 2); α = .755.

Confirmatory factor analysis (CFA) of the CSWB and subjective happiness indicates that these scales are valid as they met the required model fitting indices (S1 File).

#### 2.2.2. Fear of COVID-19 (FC).

**Fig 3:**
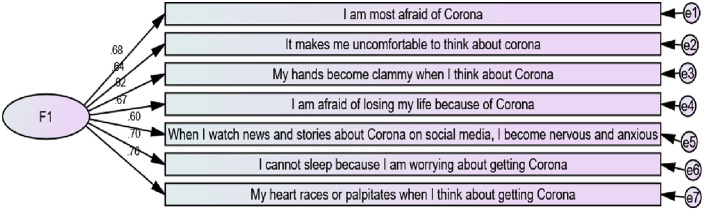
Constrained measurement model for fear of COVID-19 (F1) with standardised estimates.

Fear of COVID-19 was measured using seven items that assess how adolescents think and feel about the COVID-19 pandemic/virus [1] (See Fig 3). Participants indicated their level of agreement with each question on the scale of 1- strongly agree to 5- strongly disagree. The scale was reliable for analysis (α = .850). CFA of the scale indicates that it met some required model fitting indices, supporting its validity.

#### 2.2.3. COVID-19-induced changes in multiple life domains.

This study focuses on changes in life domains of subjective feelings of unsafety, peer relationship, and positive affect [35]. Changes in positive affect (CPA) was assessed by two items adopted from the ‘change in affect during COVID-19 scale’ [14]. The participants were asked: ‘Since COVID-19, have you felt more or less of the following: (1) ‘Happy’ and (2) ‘Joyful’? The responses were recorded on a scale of 1 = much less than before to 5 = much more than before. A higher score implies increase in positive affect while a lower score implies decrease in positive affect since the COVID-19 pandemic. The scale was reliable for analysis (α = .901). Change in peer relationship (CPR) was measured by two items adopted from the ‘change in relationship during COVID-19 scale’ [14]. The participants were asked: ‘Since COVID-19, have you noticed more or less of the following: (1) Time spent with friends and (2) Receiving support from friends? The responses were recorded on a scale of 1 = much less than before to 5 = much more than before. A higher score implies increase in peer relationships while a lower score implies decrease in peer relationships (decrease in time spent with friends and support received from friends) since the COVID-19 pandemic, α =.601. Change in subjective feelings of unsafety was assessed using a single-item measure [35] to reflect feelings of unsafety during the pandemic. This was assessed on a scale of 1- strongly agree to 5- strongly disagree by asking the participants: ‘How much do you agree with the following statement’: ‘I generally don’t feel safe since the COVID-19 pandemic’. The higher the score, the more they felt unsafe during the pandemic and vice versa.

#### 2.2.4. Sociodemographic Factors (SDFS).

Several SDFs were included as control variables, spanning personal, family and school characteristics, including sex (boys, girls and others), age, educational class level, family structure, ethnicity and religion. The participants’ knowledge of COVID-19 virus and awareness of someone infected with the virus were included as control variables as they are likely to play a role in reduction in fear of COVID-19 (Sattler et al., 2022).

#### 2.2.5. Statistical analysis-mediation model.

A mediating, or indirect relation indicates that a third variable explains the relationship between two variables [33]. In a mediated model which contains control variables and mediators, the assumptions are that the effect of fear of COVID-19 can be categorised as a direct effect of fear of COVID-19 on SWB after excluding mediated effects of the life domains (assume mediation occurring) and total effects which are the effects of fear of COVID-19 including all mediated effects of COVID-19-induced changes in peer relationship, safety and affect (assume no mediation occurring) [37]. PROCESS MACRO in SPSS was used for the mediation analysis. The analysis reported in the results section are thus explained below.

Firstly, PROCESS which is an ordinary least square and logistic regression path analysis modelling tool used an ordinary least square regression to estimate the direct effect of fear of COVID-19 on changes in the employed multiple life domains and adolescents’ SWB in the presence of the SDFs (see Table 4). The direct effect in mediation analysis measures the extent to which adolescents’ SWB alters when fear of COVID-19 increases by one unit and changes in life domain remain unchanged. Secondly, PROCESS used a path analytical framework and a bootstrapping approach to analyse the indirect effect of fear of COVID-19 on the adolescents’ SWB through changes in life domain during the pandemic. Unlike direct effects, the indirect effect measures the extent to which adolescents’ SWB changes when fear of COVID-19 is held constant while changes in life domain change by the amount it would have changed had fear of COVID-19 increased by one unit. PROCESS tested the indirect effects using a bootstrapping estimation approach with a bootstrap sample of 5,000 at a 95% bias-corrected confidence interval (95% BCCI). The bootstrapping technique provides an efficient method to ensure that the specified models are constant and robust for the analysis, boosting the precision of results.

The researchers specified all intended mediators (CPR, CPA, and CSFU) in the model based on theoretical reasoning and their mediating effects are confirmed using the lower and upper limit values of each of their confidence intervals, respectively. The endpoints of the confidence interval are defined by percentiles in the allocation of bootstrap estimates of the indirect effect [37]. The bootstrapping technique was used to analyse four separate models. *Model 1* examined the mediating role of COVID-19-induced changes in multiple life domains in the relationship between FC and OLS, while *Model 2* examined the mediating role of COVID-19-induced changes in multiple life domains in the relationship between FC and CSWB. *Model 3* examined the mediating role of COVID-19-induced changes in multiple life domains in the relationship between FC and overall happiness, while *Model 4* examined the mediating role of COVID-19-induced changes in life multiple domains in the relationship between FC and subjective happiness. The IBM-SPSS software for Windows (version 23.0) was used for all analyses. The level of statistical significance was set at *p < 0.05* (two-tailed), and unstandardised coefficients are reported (33, 34). Control variables (SDFs) were included in all empirical models.

## 3. Results

### 3.1. Descriptive statistics

Table 1 shows that the mean age of the 517 adolescents was 17.41, *SD* = ± 1.12) years, and there were more boys (59.4%) than girls (40.6%). Majority were second-year students (78.3%), and more than half of them lived with both parents (59.6%). Most of the adolescents reported that they were aware of the COVID-19 virus (33.1% a little, 23.8% some, and 35.4% a lot), and 93.4% were not personally aware of anyone who had been infected with the virus. Majority reported higher fear of COVID-19, decrease in peer relationship, positive affect and increase in feelings of unsafety. Regarding results for the dependent and independent variables, see Table 2.

**Table 1:** Demographic characteristics of participants.

Variables	Valid N	Mean (±SD)	(%)
Age (R = 14–19)		17.41 (1.12)	
**Sex**			
Boy	307		(59.4)
Girl	210		(40.6)
**Class in school**			
Form 1	108		(20.9)
Form 2	405		(78.3)
Form 3	2		(.4)
Missing	2		(.4)
**Family structure**			
Single parent	109		(21.1)
Both parents	308		(59.6)
Stepparents	9		(1.7)
Family relatives	89		(17.2)
Others	2		(.4)
**Ethnicity**			
Mole	3		(.6)
Dagbon	27		(5.2)
Grusi	6		(1.2)
Lobi	24		(4.6)
Dagao	228		(44.1)
Sissala	47		(9.1)
Waala	128		(24.8)
Others	54		(10.4)
**Religion**			
Christianity	337		(65.2)
Islam	158		(30.6)
Traditionalist	9		(1.7)
Other	6		(1.2)
Missing	7		(1.4)
**Know a COVID-19 infected person**			
No	483		(93.4)
Yes	34		(6.6)
**Knowledge about COVID-19**			
Nothing	40		(7.7)
A little	171		(33.1)
Some	123		(23.8)
A lot	183		(35.4)

*N = 517, % = sample percentage, R = Range, SD = Standard deviation*

**Table 2:** Participant characteristics and study measures.

	Mean (±SD)
Fear of COVID-19 (R = 7–35)	24.14 (6.68)
Overall life satisfaction (R = 0–10)	5.22 (3.13)
Composite subjective well-being (7–40)	22.60 (6.71)
Overall happiness (R = 1–4)	2.07 (0.97)
Subjective happiness (R = 3–21)	12.61 (4.89)
Change in peer relationship (R = 2–10)	5.11 (2.19)
Change in positive affect (R = 2–10)	4.82 (2.41)
Change in subjective feeling of unsafety (R = 1–5)	3.55 (1.37)

*N = 517, % = sample percentage, R = Range, SD = Standard deviation*

### 3.2. Associations between fear of COVID-19, COVID-19-induced changes in multiple life domains and subjective well-being

As shown in Table 3, Pearson correlation analysis revealed statistically significant but weak negative associations between fear of COVID-19 (FC) and change in peer relationship (CPR) and positive affect (CPA) but a positive association with feelings of unsafety. FC negatively correlated with all the measurements of adolescents’ SWB (overall life satisfaction-OLS, composite SWB-CSWB, overall happiness and subjective happiness). Changes in all the life domains significantly correlated with the measures of SWB except for CPR which did not correlate with overall happiness and composite SWB.

**Table 3:** Pearson correlation analysis of associations between fear of COVID-19, Changes in Life Domain and SWB of Adolescents.

	1	2	3	4	5	6	7	8
1. Overall life satisfaction	1							
2. Overall happiness	.265***	1						
3. Composite subjective well-being	.438***	.312***	1					
4. Subjective happiness	.460***	.283***	.469***	1				
5. Fear of COVID-19	−.171***	−.125**	−.087**	−.190***	1			
6. Change in peer relationship	.154***	.030	.058	.117**	−.084	1		
7. Change in positive affect	.195***	.155***	.194***	.233***	−.219***	.284***	1	
8. Change in subjective feeling of unsafety	−.137**	−.163***	−.213***	−.149**	.628***	−.117**	−.145**	1
*N*	516	517	517	517	517	516	514	514

***p<.001, **p<.005, *p<0.05, N = sample size

### 3.3. The Direct Effect of Fear of COVID-19, Change in Peer Relationship, Positive Affect and Subjective feelings of unsafety on Different Measurements of SWB during COVID-19

**Fig 4:**
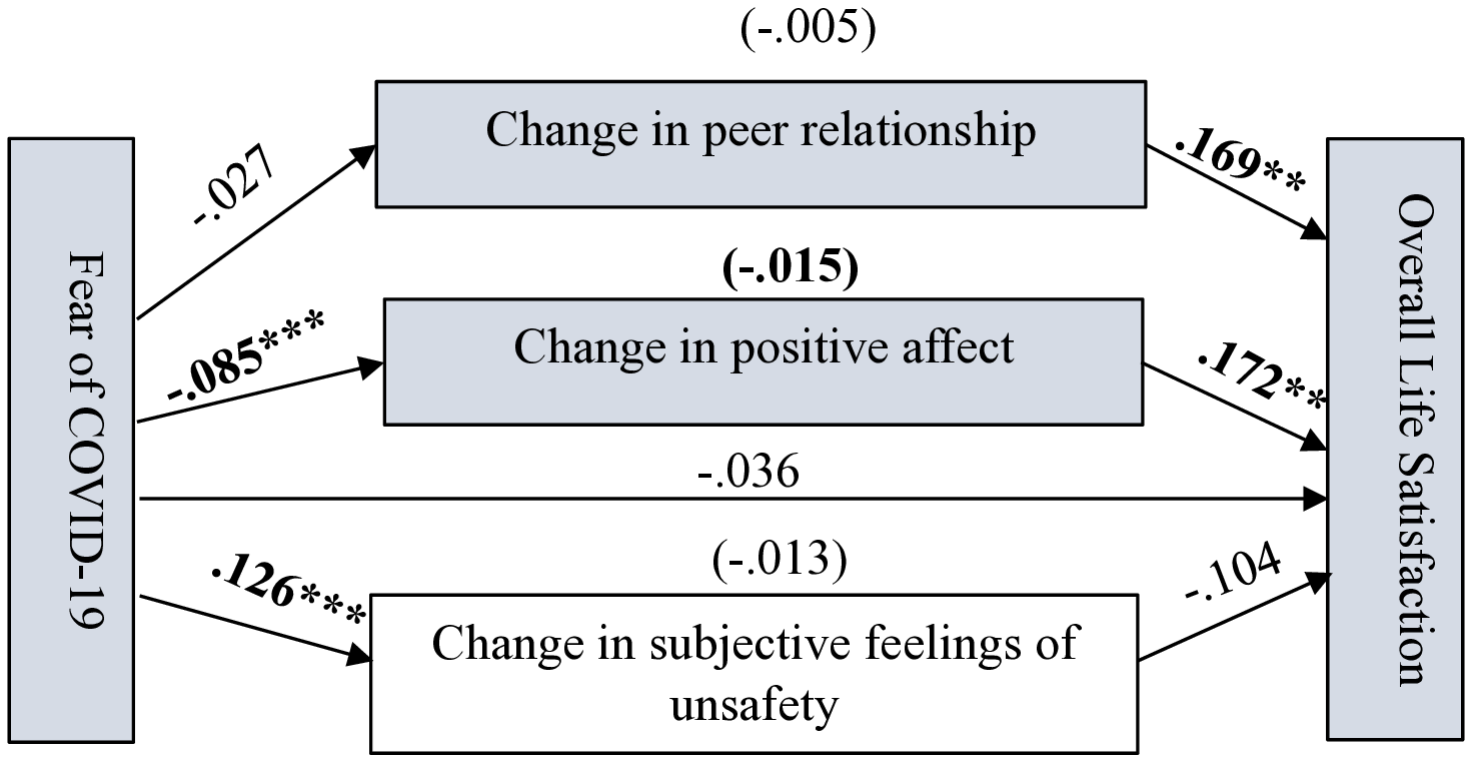
Path estimates in Model 1 showing fear of COVID-19’s pathways to adolescents’ overall life satisfaction. Significant coefficients are in bold. N = 504, ***p <.001, **p <.005.

**Fig 5:**
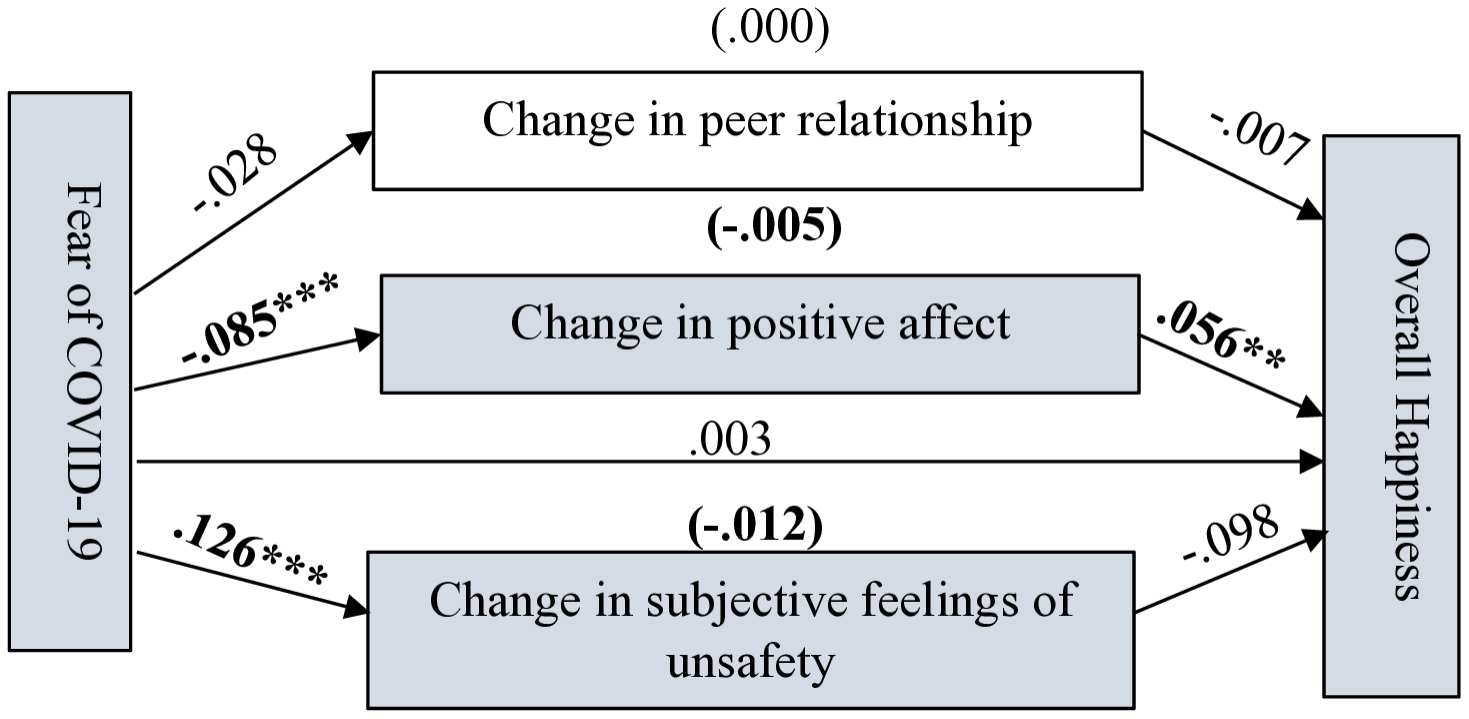
Path estimates in Model 3 showing Fear of COVID-19’s pathways to adolescents’ overall happiness. Significant coefficients are in bold. N = 505, ***p <.001, **p <.005.

**Fig 6:**
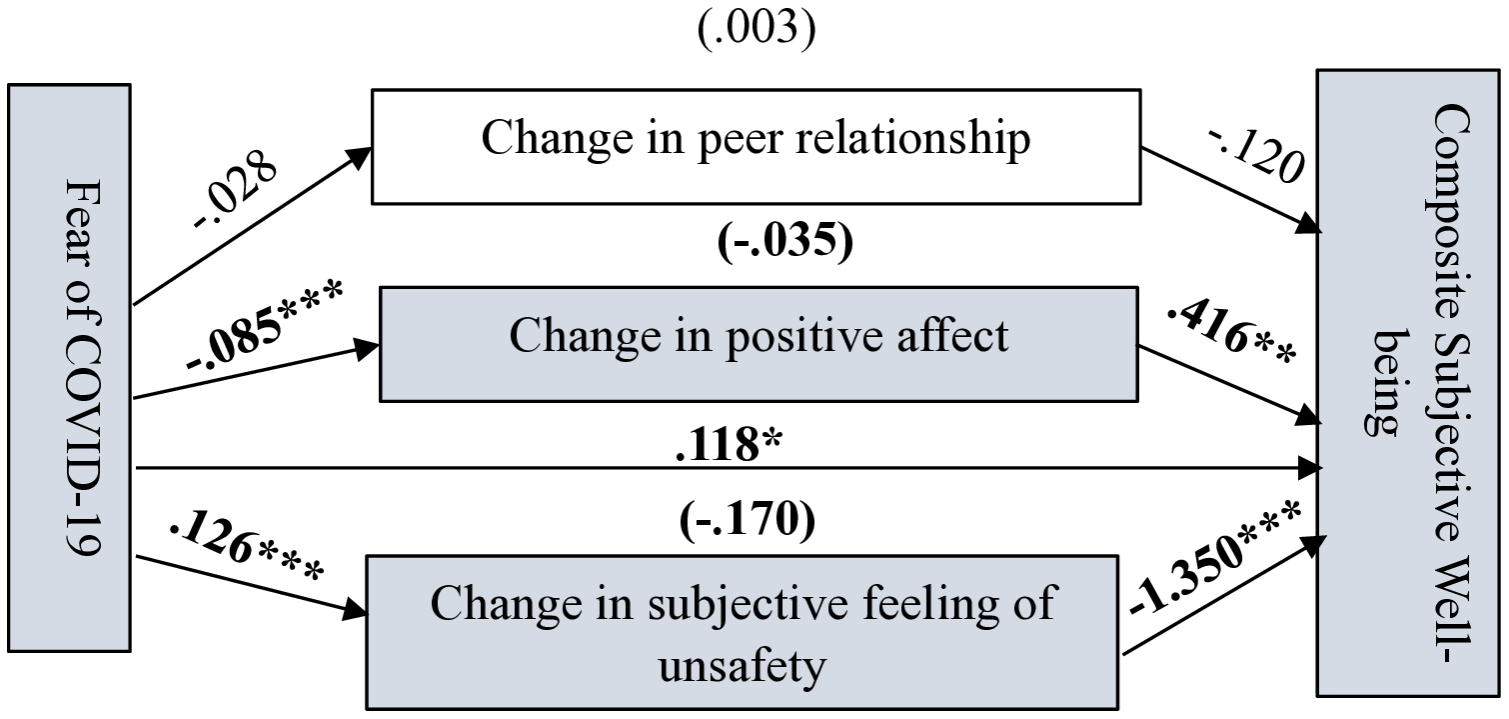
Path estimates in Model 2 showing fear of COVID-19’s psychosocial pathways to adolescents’ composite subjective well-being. Significant coefficients are in bold. N = 505; ***p <.001, **p <.005, *p <.05.

**Fig 7:**
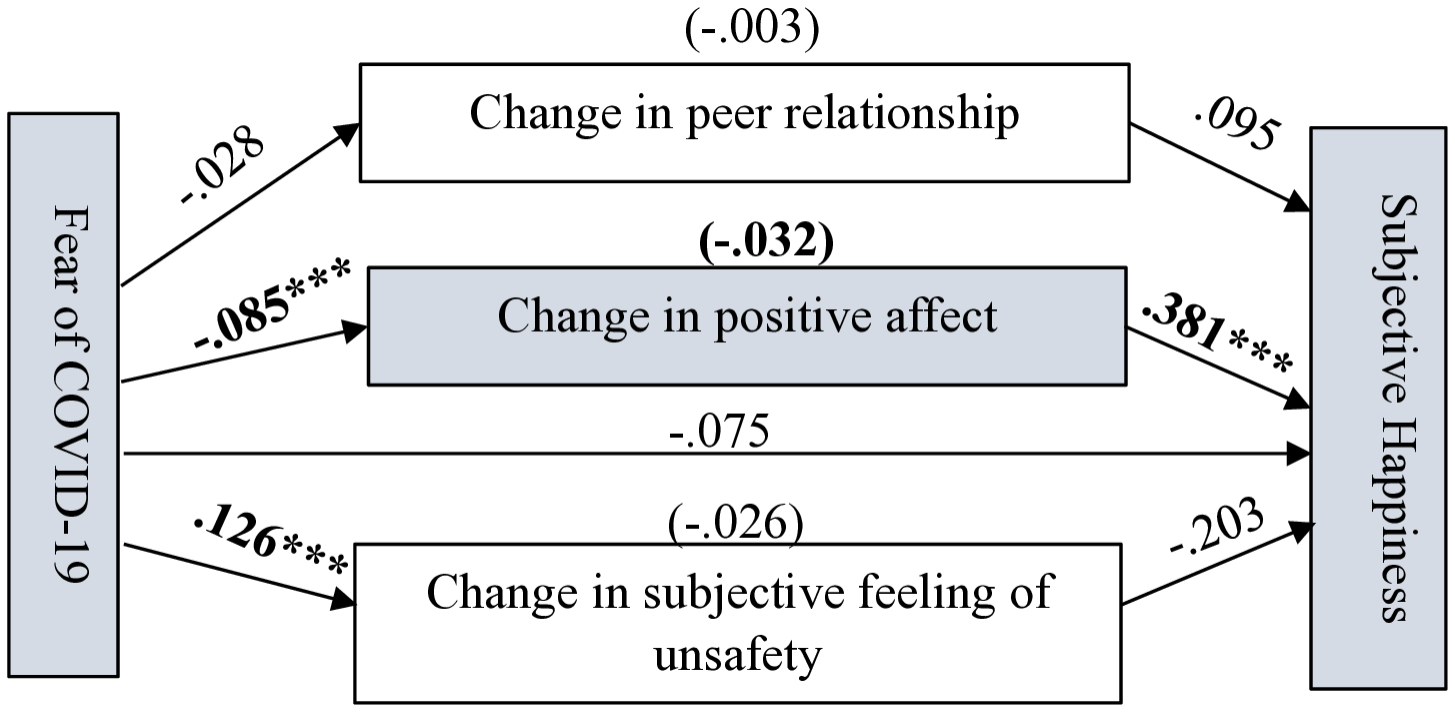
Path estimates in Model 4 showing fear of COVID-19’s pathways to adolescents’ subjective happiness. Significant coefficients are in bold. N = 505, ***p <.001.

As shown in Figs 4–7 (significant coefficients are boldened) and Table 4, on the four measurements of SWB, change in peer relationship (CPR) predicted only overall life satisfaction (OLS). Change in subjective feelings of unsafety (CSFU) predicted CSWB and overall happiness, while change in positive affect predicted all the measurements of SWB during the pandemic. Fear of COVID-19 negatively predicted CPA and positively predicted CSFU. As shown in Table 4, on the four measurements of SWB, in the absence of the mediators (total effects), FC significantly predicted OLS, subjective happiness and overall happiness. However, after accounting for the mediators, FC predicted only CSWB but in an unexpected direction which suggest possible interaction effects occurring among the variables in the model; caution is needed for interpreting this finding.

**Table 4:** Ordinary Least Squares Regression Examining Predictors of Different Measurements of SWB.

	Model 1 OLS	Model 2 CSWB	Model 3 Overall happiness	Model 4 Subjective happiness
***Independent variables***	***B* (SE)**	***B* (SE)**	***B* (SE)**	***B* (SE)**
Fear of COVID-19 (FC)	−.036 (.026)	.118 (.057)*	.003 (.008)	−.075 (.044)
Change in peer relationship (CPR)	.169 (.061)*	−.120 (.139)	−.007 (.021)	.095 (.108)
Change in positive affect (CPA)	.172 (.061)*	.416 (.131)**	.056 (.019)**	.381 (.102)***
Change in subjective feeling of unsafety	−.104 (.128)	−1.350 (.274)***	−.098 (.040)*	−.203 (.214)
***Covariates***				
Sex (Reference group- boys)	−.587 (.283)*	−1.369 (.606)*	−.208 (.089)*	−.921(.472)
Age	−.252 (131)	−.524 (.281)	−.036 (.041)	−.653 (.219)**
Knowledge of someone with COVID-19	.422 (.541)	−.019 (1.148)	.176 (.169)	−.702 (.894)
Knowledge of COVID-19	.243 (.138)	−.019 (.296)	.074 (.044)	.358 (.231)
Class level	.120 (.357)	−.874 (.766)	−.152 (.113)	.899 (.597)
Ethnicity	−.092 (.096)	−.033 (.030)	−.033 (.030)	.089 (.160)
Family Structure	.264 (.142)	.509 (.305)	.049 (.045)	−.056 (.237)
Religion	.224 (.191)	.751 (.411)	.048 (.061)	.243 (.320)
R-square (N)	.097 (504)	.117 (505)	.068 (505)	.108 (505)
*Total effects of FC*	−.068 (.021)**	−.084 (.045)	−.014 (.007)*	−.136 (.035)***

B = unstandardised coefficients, SE = standard error. ***p < .001, **p < .005, *p < 0.05. All control variables were included in the models. Significant coefficients are in bold.

*Overall life satisfaction (OLS)* As hypothesised, adolescents who reported decrease in peer relationship and positive affect during the pandemic were more likely to experience lower OLS than their colleagues who reported increase in both peer relationship and positive affect during the pandemic. Although the total effect of FC on OLS was significant (c = −.068, SE = .021, 95% CI [−.109, −.027], p < .005), the direct effect of FC on OLS was not significant after controlling for the mediators (c’ = −.036, SE = .026, 95% CI [−.087,.016], *p* > .05). The assumption that FC will predict OLS was hence not supported. This suggests possible mediation occurring in Model 1 (See Fig 4). The model summary shows that all the employed variables significantly predicted OLS (R = .311, R-sq = .097, MSE = 8.924, F = 4.396, df1 = 12.000, df2 = 491.000, *p* = .000).

*Overall happiness* Again, as shown in Fig 5 and Table 4, adolescents who reported decrease in positive affect stood higher chances of experiencing lower overall happiness than those who reported increase in positive affect during the pandemic. Adolescents who reported an increase in feelings of unsafety were more likely to experience lower overall happiness. Although the correlation results revealed a significant negative association between FC and overall happiness, the effect on overall happiness was insignificant once the potential mediators and sociodemographic controls were accounted for in Model 3. Although the total effect of FC on overall happiness was significant (c = −.014, SE = .007, 95% CI [−.027, −.001], *p* < .05), the direct effect of FC on overall happiness was not significant after controlling for the mediators (c’ = .003, SE = .008, 95% CI [−.014,.019], *p* > .05). The model summary shows that all the employed variables significantly predicted overall happiness (R = .261, R-sq = .068, MSE = .895, F = 3.000, df1 = 12.000, df2 = 492.000, *p* = .000).

*Composite SWB (CSWB) and subjective happiness* From Fig 6 and Table 4, adolescents who reported decrease in positive affect and increase in feelings of unsafety were more likely to report decrease in CSWB and vice versa. The total effect of FC on CSWB was negative but not significant (c = −.084, SE = .045, 95% CI [−.174,.005], *p* > .05), however, after accounting for the mediators, the direct effect of FC on CSWB was significantly positive (c’ = .118, SE = .057, 95% CI [.007,.229], *p* < .05).

Lastly, as shown in Fig 7, the findings indicate that adolescents who reported an increase in positive affect were more likely to report higher happiness compared to those who reported a decrease in positive affect during the pandemic. The total effect of FC on subjective happiness was significant (c = −.136, SE = .035, 95% CI [−.204, −.067], *p* < .001) but after controlling for the mediators the direct effect of FC on subjective happiness was insignificant (c’ = −.075, SE = .044, 95% CI [−.162,.012], *p* > .05). The model summary shows that all the employed variables significantly predicted CSWB (R = .341, R-sq = .117, MSE = 41.175, F = 5.415, df1 = 12.000, df2 = 492.000, *p* = .000) and subjective happiness (R = .321, R-sq = .103, MSE = 24.972, F = 4.720, df1 = 12.000, df2 = 492.000, *p* = .000).

Regarding the SDFs, sex predicted OLS, CSWB and overall happiness indicating poorer outcomes for the adolescent girls while age negatively predicted subjective happiness. Having knowledge of someone infected with COVID-19 and knowledge of the COVID-19 virus had no significant effects on the different measures of SWB.

### 3.4. Indirect Effects of Fear of COVID-19 on Different Measurements of SWB through Change in Peer Relationship, Positive Affect and Subjective feelings of unsafety during COVID-19

As hypothesised, changes in subjective feelings of unsafety, peer relationship and positive affect mediated the effect of FC on different measurements of SWB, showing varying effects. This likely explains why the direct effect of FC on the different measurements of SWB were insignificant after including the mediators in the models. As shown in Table 5 and Fig 4, the indirect effect of FC on OLS through CPA was −.015 (95% CI [−.029, −.004]) and through CPR was −.005 (95% CI [−.012,.000]). CSFU could not mediate the effect of FC on the adolescents’ OLS. The total indirect effect was −.032 (95% CI [−.071,.001]). Again, as shown in Figs 6 and 7and Table 5, the indirect effect of FC on CSWB through CPA was −.035, (95% CI [−.065, −.012]) and through CSFU was −.170, 95% CI [−.245, −.102]). The total indirect effect was −.202 (95% CI [−.281, −.134]). The indirect effect of FC on subjective happiness through CPA was −.032 (95% CI [−.057, −.012]). CPR and CSFU could not mediate the effect of FC on subjective happiness. The total indirect effect of FC on subjective happiness through the mediators was −.061 (95% CI [−.121, −.002]).

**Table 5:** Bootstrapping Mediation Analysis in Model 1-Model 4: Indirect Effect of Fear of COVID-19 (FC) on SWB Through Change in Peer Relationship (CPR), Positive Affect (CPA) and Subjective Feelings of Unsafety (CSFU).

Indirect paths of fear of COVID-19	*B* (SE)	BCCI
*Model 1-Overall Life Satisfaction (OLS) (N = 504)*		
FC → CPR → OLS	**−.005 (.003)**	**[-.012,.000]**
FC → CPA → OLS	**−.015 (.006)**	**[-.029, -.004]**
FC →CSFU→ OLS	−.013 (.017)	[-.050,.018]
*Model 2-Composite Subjective Well-Being (CSWB) (N = 505)*		
FC → CPR →CSWB	.003(0.005)	[-.004, 0.015]
FC → CPA→CSWB	**−.035 (0.013)**	**[-.065, -0.012]**
FC →CSFU→ CSWB	**−.170 (0.037)**	**[-.245, -0.102]**
*Model 3-Overall Happiness (OH) (N = 505)*		
FC → CPR → OH	.000 (0.001)	[-.001,.002]
FC → CPA → OH	**−.005 (0.002)**	**[-.009, -.001]**
FC →CSFU→ OH	**−.012 (0.005)**	**[-.023, -0.002]**
*Model 4-Subjective Happiness (SH) (N = 505)*		
FC → CPR → SH	−.003 (0.004)	[-.012,.005]
FC → CPA → SH	**−.032 (0.011)**	[-**.057, -.012]**
FC →CSFU→ SH	−.026(0.028)	[-.083,.027]

*B = unstandardised coefficients, SE = bootstrapping standard error, BCCI = bias-corrected confidence intervals. All the sociodemographic variables were controlled for in the models. OLS = Overall Life Satisfaction, CSWB = Composite SWB, OH = Overall Happiness and SH = Subjective Happiness. Significant coefficients are in bold.*

Referring to Fig 5 and Table 5, the indirect effect of FC on overall happiness through CPA was −.005 (95% CI [−.009, −.001]) and through CSFU was −.012 (95% CI [−.023, −.002]). The total indirect effect of FC on overall happiness through the mediators was −.017 (95% CI [−.028, −.006]).

The results overall suggest that changes in positive affect (CPA) was the strongest mediating variable, as it mediated the effects of Fear of COVID-19 on all the measurements of SWB.

## 4. Discussion

While many studies on the impact of COVID-19 pandemic’s effects on individuals’ life domains and quality of life exist, there remains unanswered questions on the mechanisms that explain the impact of fear of COVID-19 on different measurements of adolescents’ subjective well-being particularly in the sub-Saharan African context. Answering the research questions and testing the hypotheses using the life event theory and the Stress, Emotions, and Performance meta-model, this study has shown that indeed the COVID-19 pandemic’s effects, particularly, fear of COVID-19 and COVID-19 induced changes in peer relationships, subjective feelings of unsafety and positive affect have posed significant consequences to the SWB of adolescents in the sub-Saharan African region, Ghana. We found that many adolescents from Ghana experienced high fear, reduction in their sense of safety, peer relationship, as well as reduction in positive affect and SWB due to the pandemic. The adolescent girls were particularly left behind in all dimensions and measurements of SWB during the pandemic as found in an adult study [18]. By employing multiple life domains and different measurements of SWB, we revealed variations in the impact of the pandemic’s effect, fear of COVID-19, on different life domains and SWB of adolescents from Ghana. The study’s findings suggest that the life event theory is supported by this study, as we found that the pandemic was indeed a negative life event as fear of COVID-19 directly and negatively affected various life domains of the adolescents including their quality of life. Also, the findings support the Stress, Emotions, and Performance meta-model as we found that the effect of fear of covid-19 which was a stressor in this study was mediated by the adolescents’ appraisal of changes in their life experiences during the pandemic which resulted in varying experiences of subjective well-being. These changes in their life experiences were mediators that functioned as either coping mechanisms or risk/protective factors for their subjective well-being against effects of fear of COVID-19. For instance, negative changes in feelings of safety such as high feelings of unsafety acted as a risk factor while positive changes such as high peer relationships and positive affect during the pandemic acted as protective factors for the adolescents’ overall life satisfaction, composite subjective well-being, subjective happiness and overall happiness.

### 4.1. Association between Fear of COVID-19, COVID-19-Induced Changes in Multiple Life Domains and SWB

Findings from the correlation analysis revealed that experiencing high fear of COVID-19 was associated with adolescents reporting decrease in positive affect and increase in feelings of unsafety during the pandemic. This mirrors exiting studies that found that fear of COVID-19 was associated with changes in social and psychological experiences during the pandemic [4,6,17,38]. If the adolescents became afraid of catching or spreading the virus, it could trigger negative emotions and a sense of unsafety in their environments thereby reducing positive affect. Again, irrespective of the type of measurement of SWB employed, high fear of COVID-19 was associated with adolescents reporting poorer SWB including lower life satisfaction and happiness as reported from adult studies [4,17,23]. This could be related to the negative psychological impact of fearof-COVID-19 such as psychological distress, mental health impairment, anxiety and depression on adolescents [4,9,18]. Moreover, as proposed by the life event theory, adolescents that experienced negative changes in peer relationship, positive affect and subjective feelings of unsafety during the pandemic experienced poorer SWB irrespective of the employed measurements of SWB including life satisfaction and happiness. This could be because such adolescents lost crucial socioemotional resources such as feelings of safety, positive affect and strong peer relationships needed to boost their SWB during the pandemic [6,7,38].

### 4.2. Direct Effects of Fear of COVID-19 and COVID-19-Induced Changes in Multiple Life Domains on SWB

As hypothesised and proposed by the life event theory, the regression analysis revealed that indeed, fear of COVID-19 contributed negatively to the induced changes in adolescents’ positive affect and subjective feelings of unsafety during the pandemic by reducing positive emotions and feelings of safety. Adding to the earlier explanation of the Stress, Emotions, and Performance meta-model, this could be related to stress response from fear of COVID-19 such as psychological distress and anxiety associated with fear of COVID-19 [4]While the effect sizes are not large, these effects remain significant and warrant discussion due to the possible long-term consequences these negative psychological experiences could have on adolescents’ mental health post-pandemic [7]. Surprisingly, fear of COVID-19 did not influence significant changes in the adolescents’ peer relationship; this could be because the participants were living in school dormitories with their peers during the survey and so they did not recognise much differences in their peer relationships during the pandemic. Furthermore, as emphasised by the life event theory and the Stress, Emotions, and Performance meta-model, changes in peer relationship, positive affect, and subjective feelings of unsafety generally contributed to the variations in adolescents’ SWB including life satisfaction and happiness during the pandemic after controlling for demographic factors. Adolescents who responded negatively to the pandemic such as experiencing decrease in peer relationship and positive affect and increase in subjective feelings of unsafety were more likely to experience poorer SWB including lower happiness and lower life satisfaction and vice versa. These changes imply that the adolescents experienced significant reduction in crucial psychosocial resources that are necessary for boosting their SWB during the pandemic [6,7]. Existing studies have revealed disruptions to social relationships as a culprit for the prevalent deterioration in mental health and well-being of young people during the pandemic [6,7]. A reduction in positive affect during the pandemic might have resulted in increased negative affect such as anxiety, stress, depression and loneliness among the participants; resulting in poorer SWB [2,4,5]. Additionally, feelings of unsafety have been associated with psychological distress [27] and depression and among adolescents during the pandemic [38].

The findings again revealed that, similar to existing studies, there are direct effects of fear of COVID-19 on the different measurements of SWB including life satisfaction and happiness [4,17,22,23] in the absence of the mediators (changes in ‘peer relationship’, ‘subjective feelings of unsafety’ and ‘positive affect’). However, contrary to the study hypothesis, we did not find significant direct negative effects of fear of COVID-19 on the different measurements of SWB in the presence of the mediators. This variation could be due to the fact that, the effect of fear of COVID-19 on SWB of adolescents was indirect and that ‘changes in peer relationship’, ‘subjective feelings of unsafety’ and ‘positive affect’ completely mediated the effects of fear of COVID-19 on the different domains and measurements of SWB (life satisfaction and happiness) (supported in the mediation analysis). Surprisingly, however, the direct effect of fear of COVID-19 on one measurement of SWB, composite SWB became positive after accounting for changes in peer relationship, subjective feelings of unsafety and positive affect. While interpretation of this finding should be done with caution, this finding could be related to some of the unexpected positive effects of the pandemic that have been reported across studies involving young people such as improved relationships, improved health and SWB during the pandemic [40,41]. This is likely because, due to fear of COVID-19 infection, the adolescents might have spent more time at home with family, building family bonds that could consequently enhance their SWB.

Findings from the direct effect of fear of COVID-19 on varying measurements of SWB of adolescents support the theoretical underpinning of this study and suggest the important role of negative life events in directly influencing adolescents’ life experiences and psychological well-being. It implies the need for researchers to evaluate both the cognitive and hedonic components of SWB as life events have varying impacts on varying dimensions of adolescents’ outcomes.

### 4.3. Indirect Effects of Fear of Covid-19 on SWB

Supported by existing studies, the life event theory and the Stress, Emotions, and Performance meta-model have revealed the answer to the research question ‘What mechanism link COVID-19 induced changes in multiple life domains to fear of COVID-19 and SWB among adolescents in SSA?’. The findings offer an explanation to the ‘why’ and ‘how’ questions concerning the impact of fear of COVID-19 on the SWB of adolescents in SSA. The findings from the mediation analyses affirm negative indirect effects of fear of COVID-19 on the different measurements of SWB [4,17] and that the variations in the effect of fear of COVID-19 on the adolescents SWB were due to variations in the life experiences (changes in peer relationship, subjective feelings of unsafety, and positive affect) of the participants. The employed mediation models revealed negative indirect effects of fear of COVID-19 on SWB, in that changes in positive affect mediated the relationship between fear of COVID-19 and all the different measurements of SWB (overall life satisfaction, overall happiness, subjective happiness and composite SWB) of the participants. Thus, changes in positive affect during the pandemic buffered and protected some adolescents from experiencing negative consequences of fear of COVID-19 on their SWB. This could be because when adolescents experience an increase in positive affect, it could help them to experience good feelings like joy and happiness which could help build resilience against psychological distress caused by fear of COVID-19 and consequently safeguarding their SWB. Again, changes in peer relationship mediated the relationship between fear of COVID-19 and one measurement of SWB (overall life satisfaction) showing negative indirect effect of fear of COVID-19 on SWB. Thus, adolescents who experienced increase in peer relationship were likely to have received high support from friends and spent adequate time with friends which could enhance their access to psychosocial resources needed to build resilience [34] against effects of fear of COVID-19, thereby consequently protecting their life satisfaction from fear of COVID-19’s negative effects. Peer relationship has been found to function as a protective health asset that buffers the negative effects of life circumstances on adolescents’ SWB [34]. Moreover, changes in subjective feelings of unsafety mediated the relationship between fear of COVID-19 and two measurements of adolescents’ SWB (Composite SWB and overall happiness) showing that decrease in feelings of unsafety safeguarded the adolescents SWB amidst fear of COVID-19’s negative effects. Findings show that the safer individuals feel in their neighbourhood, the less psychological distress they experience [36], hence, increase in feelings of safety could have buffered psychological distress associated with fear of COVI-19 and consequently boosted their SWB. Similarly, feelings of safety have been reported as a mediator and protective factor against depression among adolescents during the pandemic [39]). This suggests a negative indirect effect of fear of COVID-19 on SWB through subjective safety.

Findings from the mediation analyses offer significant theoretical contributions to the existing literature in that there is a need to recognise that adolescents cope and respond differently to the same life event, and that for some adolescents experiencing positive changes during the pandemic helped protect their SWB against negative impacts of fear of COVID-19. Aligning this with the life event theory and the Stress, Emotions, and Performance meta-model, the role of public health interventions that offer coping mechanisms and protective factors for adolescents could be explored by researchers to develop appropriate intervention programmes that could protect adolescents during pandemics and protect their healthy development post-pandemic. Employing the Stress, Emotions, and Performance meta-model particularly can provide insight into how adolescents respond to and cope with negative live events or stressors from their environment to protect their subjective well-being. This can help to explain the how and why some adolescents fare well during a negative life event while others do not. Thus, help to explain the mechanisms that link stressors/life events to the quality of life of adolescents.

### 4.4. Strengths and limitations

This study has contributed to knowledge by providing robust empirical evidence on the effect of fear of COVID-19 on multiple life domains of safety (subjective feelings of unsafety), emotional well-being (positive affect), intimacy (peer relationship) and SWB of adolescents. The current study again makes significant contributions to research and ongoing scientific discussion about the mechanisms linking the COVID-19 pandemic’s effects to individual’s quality of life (SWB) as well as the impact of the pandemic’s effects on different measurements of SWB especially in the sub-Saharan African context. This study is the first to employ the life event theory and the Stress, Emotions, and Performance meta-model to provide evidence on the ‘mediating’ mechanism that links fear of COVID-19 and COVID-19 induced changes in domains of feelings of unsafety, positive affect and peer relationships to different measurements of adolescents’ SWB (overall life satisfaction, overall happiness, subjective happiness, and composite subjective well-being). By simultaneously examining the impact of fear of COVID-19 on multiple life domains and different measurements of SWB, we make methodological contributions to promote holistic investigation into the impact of the pandemic’s effects on young people’s quality of life as well as understanding of the mechanisms that could explain the relationship between fear of COVID-19, COVID-19 induced changes in life domains and SWB of adolescents. Despite these strengths, the weaknesses of the study include its inability to establish causality among the variables due to the use of cross-sectional survey and the limited sample size used due to COVID-19 restrictions at the time of the data collection. Also, due to lack of many existing studies related to the relationships been explored in this study, we could not employ a priori power analysis to estimate the sample size. Again, self-assessment scales were used in the questionnaire which could introduce some bias in the responses; however, a follow up focus group discussion was done after the survey to validate the responses from the questionnaire hence reducing bias in the data. Despite all these limitations, this study provides rigorous and novel insights into the mechanisms and impacts of COVID-19-related effects and young people’s SWB. Future studies could consider longitudinal study using a larger sample to investigate the long-term interplay between fear of COVID, COVID-19 induced changes in life domains of safety, peer relationship and positive affect and the different measurements of adolescents’ SWB. Future studies should consider the role of gender in the relationship between fear of COVID-19, COVID-9 induced changes in life domains and adolescents’ SWB.

## 5. Conclusion

While the pandemic is currently not a global emergency, these findings remain critical for discussions on the impact of the pandemic’s effects on young people’s quality of life, positive adolescent development post-pandemic, and lessons for addressing public health crises that could impact the quality of life of young people. The findings infer the need for post COVID-19 pandemic programmes and interventions from governments and NGO’s that could help adolescents to accumulate all the socioemotional resources that they lost to the pandemic. In schools, families and communities, social and mental health support should be freely available to adolescents who are still recovering from psychological distress experienced due to fear of COVID-19 and the negative changes experienced in their peer relationships, positive affect, safety and SWB. The findings also contribute to the discussion on explaining the relationship between the pandemic’s effect (fear of COVID-19) and different measurements of quality of life and SWB. Public health and intervention providers should hence recognise peer relationship, positive affect and feelings of safety as crucial protective factors that can be incorporated as complements in public health programmes that seek to protect adolescents from related long-term impact of the pandemic’s effects such as anxiety post the pandemic. For instance, in families, schools and communities, adolescent-centred programmes and activities that foster peer relationships, positive affect and feelings of safety can help affected adolescents who are especially going through post-traumatic stress disorder (PSTD) due to high anxiety and psychological distress associated with high fear of the COVID-19 and possible loss of significant relationships and positive affect during the pandemic; to improve their quality of life and SWB including life satisfaction and happiness. The lesson is for authorities to provide mitigation measures that do not negatively impact life domains of intimacy (peer relationships/social connectedness), emotional well-being (including positive affect) and safety of young people during a public health crisis. Lastly, it is, vital for policymakers, intervention providers, epidemiologists, and researchers to ensure holistic assessment of the pandemic’s impact on young people by simultaneously considering multiple domains of life as well as different measurements of quality of life including SWB.

## Supporting information

S1 File Results of confirmatory factor analysis. (PDF)
