# Supplementary material for: A mediation model explaining the impact of fear of COVID-19 and COVID-19- induced changes in multiple life domains on adolescents’ subjective well-being in sub-Saharan Africa
Source: PLoS One. 2025 Nov 19;20(11):e0337188. doi: 10.1371/journal.pone.0337188 (PMC12629459; doi:10.1371/journal.pone.0337188)
Supplement: S1 File — (PDF) [file pone.0337188.s001.pdf]

Results of Confirmatory Factor Analysis

Confirmatory Factor Analysis of Composite Subjective Well-being and Subjective Happiness

Confirmatory factor analysis (CFA) of the CSWB and subjective happiness were carried out and the findings indicate that these scales are valid as they met the required model fitting indices (RMSEA = .070, CFI = .951, NFI = .934, TLI =.916, IFI = .952).

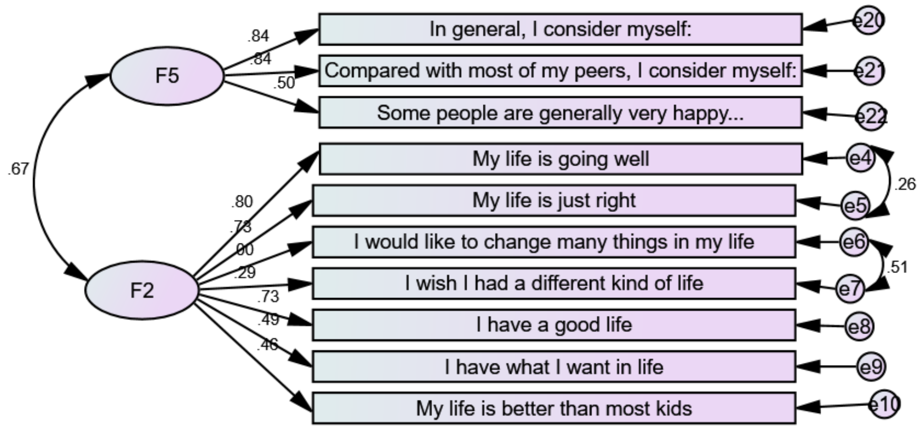

Fig 1. Constrained measurement model for subjective happiness (F5) and composite subjective well-being (F2) with standardised estimates.

Confirmatory Factor Analysis of Fear of COVID-19

CFA of the Fear of COVID-19 scale was done and the findings indicate that it met most of the required model fitting indices, supporting its validity (RMSEA = .098, CFI = .943, NFI = .933, IFI = .944, TLI = .916).

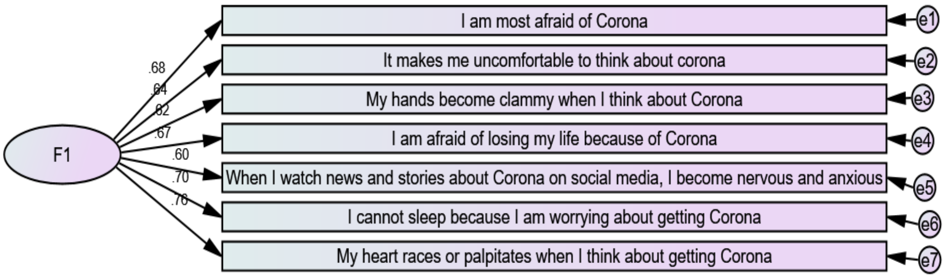

Fig 2. Constrained measurement model for fear of COVID-19 (F1) with standardised estimates.
